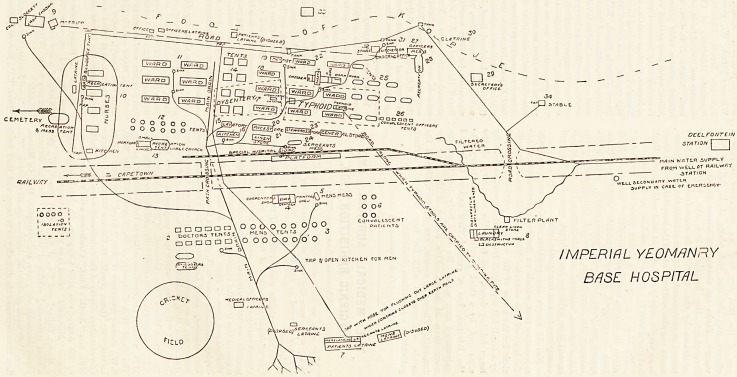# The Imperial Yeomanry Hospital

**Published:** 1900-06-23

**Authors:** Alfred D. Fripp

**Affiliations:** Senior Surgeon I. Y. H.


### The Institutional Workshop.

### THE IMPERIAL YEOMANRY HOSPITAL.

By Alfred D. Fripp, M.S., F.R.C.S., Senior Surgeon I. Y. H.

Though there are still two of the English huts unfinished, this hospital has in all other respects reached, at last, its full state of development, and a short account of some of the main features may probably be of interest. The appended plan of the hospital grounds has been prepared by Dr. Barclay Black, and with that plan in front of me, I have jotted down a description of the various parts of the hospital as one meets them in taking any visitor for a tour of inspection. This doctor, who is my staff officer, superintends all the work of the camp cleanliness and latrine departments, but his plan, not being the work of a skilled surveyor, does not pretend to be of more than approximate accuracy as to distance, but it is at least faithful in intention and in effect. To take first the smallest half, which lies to the right of the railway line as you arrive from Cape Town, the following departments are seen :—

No. 1. The group of isolation tents, for, like other base hospitals, we have not been exempt from the occurrence of an occasional case of scarlet fever.

No. 2 indicates the position of the tents of the medical staff, the two marquees being occupied by the ten surgeon's dressers.

No. 3 shows the bell tents occupied by the detachment of about 100 orderlies. They are seen to be separated by the main drain, and also by the main road (which leads from the wards to the main latrine, labelled 7), from the doctor's tents. The main drain is made of four-inch earthenware pipes, and is fed by various sinks throughout the hospital grounds. No solid matter is allowed to be thrown into the sinks, nor infected water from the enteric department (this is all carried by our natives along the route indicated not far from the laundry to the dumping ground), nor greasy water from the kitchens (this is used to feed the pigs), nor does the drain go near any latrine, nor any water-supply pipe. Practically, the only thing thrown down the sinks is water that has been used for washing. The main drain discharges by means of a system of sub-dividing trenches on to a part of the veldt which has a good incline away from the hospital.

Between No. 3 and the railway line is placed a galvanised iron building, which is subdivided into a central dry canteen for the detachment, and two lateral compartments forming workshops for the carpenters and painters respectively.

No. 5 is a large tent used as a mess-room, recreation-room, and bath-room by the detachment.

No. 6 represents a collection of hospital marquees, which are used only when the hospital is over full for the temporary accommodation of convalescent patients waiting a train for transfer to Cape Town.

No. 7 is the main urinal and latrine with 37 seats. It has separate compartments for the sergeants, the detachment, and the hospital patients; and we consider it a masterpiece for the Karroo. The floor is cemented throughout, and for four feet outside the corrugated iron walls it is inclined and grooved so efficiently that the four natives, who are perpetually on duty there, can, when they are not busy carrying buckets away to the dumping ground, frequently flush out the whole enclosure by means of an armoured hose which has a good head of water from a stand-pipe. The washings are collected in a cement-lined pit just outside, and are thence removed in buckets to the dumping ground. No buckets come back from the dumping ground to any part of the hospital without having undergone a prolonged soaking and subsequent thorough cleansing in a large tank of disinfectant at the dumping ground. The bedpans and the urine receivers from all patients in the hospital who are not able to walk to this latrine are carried thither by the orderlies and left in a special annexe, clean utensils being taken back to the wards in their place. The excreta are removed to the dumping ground by the natives from six a.m. For the enteric and dysentery departments special disinfecting latrines are provided, as will be described later on.

No. 8 is the situation of the laundry, close by which are: (*a*) a Washington Lyons Disinfector, capable of dealing with nine mattresses and many smaller articles in each batch; (*b*) a Russian bath for the treatment of stiff limbs, which are so frequent both from rheumatism and from injury; (*c*) a blacksmith's forge; (*d*) a refuse destructor, through which all paper, straw, small boxes, empty canned-meat tins, and other camp litter are passed—the ash is then buried; (*e*) a large filter plant, by Defries, driven by steam from the laundry, and large enough to cope easily with all the drinking water of the hospital.

The laundry plant is a large and efficient one by the same firm, and is worked by men sent out by that firm, helped by four of the wardmaids and some 15 natives. It easily deals with the washing of the 700 white individuals at present in our camp. All of this plant is housed in corrugated iron sheds.

All mattresses, bedding, and linen from the enteric and the other infected wards are put through the Washington Lyons Disinfector at seven o'clock in the morning, having been stored, if necessary, over night in an annexe of the enteric latrine between the enteric huts.

The last point of note is the secondary well, which has been sunk on the way to the station as a standby in case of emergency; but this water supply we have not yet had to fall back upon, as that from the railway well at the station has proved amply sufficient for our needs.

If we now retrace our steps to the situation of the main drain, and walk up a slight natural incline to the railway line, and cross the latter at the most southerly of the three level crossings indicated in the plan, and, starting from the extreme south of the hospital grounds, walk northward among the wards we shall be going up a slight hill nearly all the way. The cemetery, a quarter of a mile off towards Cape Town, lies at our back.

On our left hand side is a group of three private residences numbered 9 on the plan.

No. 10 indicates the Sisters' quarters, consisting of: (*a*) Four huts. The upper two, which are the largest, are occupied by the 29 day sisters, the next one by the 10 night sisters, and the fourth by the 10 wardmaids and the housekeeper, Miss Cheesman, whose services have been of the utmost value, for she not only attends to all the domestic arrangements of this department, but she also superintends the laundry, which, in itself, is a very onerous and important task in view of our isolated position with regard to the rest of civilisation; (b) the kitchen, which is seen close to the railway. (c) The luggage tent, placed between the two upper huts, and opening into (d) the bath tent, which has its own supply of cold water from the main and of hot water from a boiler placed half way between 9 and 10, as well as its own sink just outside the tent for discharge into the main drain. The interior of the tent is separated by curtains into several bathrooms and the floor is boarded. (e) The latrine, just behind the bath tent, is, like all the latrines in the hospital, worked on the bucket and earth system, and served by natives, who withdraw the buckets by means of a trap-door at the back of the seats without going into the latrines.

No. 11 is a group of six large huts sent out from England, with one of their foremen to put them up, from Messrs. Boulton and Paul, of Norwich. These huts are a great success. They are admirably suited to the climate, which yields considerable extremes of temperature in twenty-four hours, and they are well ventilated. The material employed is corrugated iron outside, wood inside, and felt in between. Each will be provided with a verandah as soon as our carpenters have time to get to work on the many similar improvements required throughout the hospital. Hitherto they have all been busy with the absolute essentials of construction. Each hut accommodates 34 patients without any crowding. The middle one of the first row that we encounter is full of officers, and the one just above it is full of yeomanry. Sinks are shown contiguous to all the huts throughout the hospital, and the water supply is drawn by the orderlies in cans from stand-pipes placed at convenient distances outside the hut. Boiler-tanks are also scattered about the hospital, so that there is an ample supply of both hot and cold water. Each hut has a lean-to outside for washing-up purposes.

No. 12 is a group of a dozen tents, half "tortoise" green in colour, and half "hospital" marquee. Each tent is equipped to accommodate eight iron-frame spring mattress bedsteads permanently, with which luxuries the hospital is provided throughout by the Birmingham bedstead manufacturers, but each tent is in addition liable to have thrust upon it in times of emergency four or five trestle beds.

No. 13 shows the position of the large patients' recreation tent, which on Sunday is transformed into the body of the church. The chancel, which is sufficient for the needs of the week-day services, is formed by a small hut separated from the recreation tent by large folding doors, and from the mortuary, at the altar end, by a thick partition. The mortuary, like the latrines, has a cement floor and a table and galvanised iron walls. No dead body lies there for more than 24 hours. Five o'clock is the hour for funerals. These, of course, are military, though the salute is dispensed with for fear it should disturb patients in a precarious condition.

(*To be continued.*)

## Figures and Tables

**Figure f1:**